# C-Reactive Protein Levels and Cognitive Decline following Acute Ischemic Stroke: A Systematic Review and Meta-Analysis

**DOI:** 10.3390/brainsci13071082

**Published:** 2023-07-17

**Authors:** Likun Wang, Lining Yang, Haiyan Liu, Juncai Pu, Yi Li, Lu Tang, Qing Chen, Fang Pu, Dingqun Bai

**Affiliations:** 1 Department of Rehabilitation Medicine, The First Affiliated Hospital of Chongqing Medical University, Chongqing 400016, China; W1252254516@163.com (L.W.); yangln1990@163.com (L.Y.); liuhy987167@163.com (H.L.); liyworki@163.com (Y.L.); tl19970123@163.com (L.T.); cqcq20123634@163.com (Q.C.); 2 Department of Neurology, The First Affiliated Hospital of Chongqing Medical University, Chongqing 400016, China; pujuncai0910@163.com; 3 Beijing Advanced Innovation Center for Biomedical Engineering, School of Biological Science and Medical Engineering, Beihang University, Beijing 100191, China

**Keywords:** cognition, C-reactive protein, inflammatory factor, meta-analysis, cognitive decline, ischemic stroke

## Abstract

Cognitive decline (CD) is devastating with a high incidence in patients after stroke. Although some studies have explored underlying associations between C-reactive protein (CRP) levels and cognitive decline after stroke, consistent results have not been obtained. Therefore, this meta-analysis aimed to explore whether or not higher levels of C-reactive proteins were associated with an increased risk of cognitive decline after stroke. To this end, PubMed, Embase, the Cochrane Library, and Web of Science were searched for eligible studies, and pooled effect sizes from eligible studies were calculated using random effect models. Furthermore, subgroups were established and meta-regression analyses were performed to explain the causes of heterogeneity. Eventually, nine studies with 3893 participants were included. Our statistical results suggested that the concentrations of peripheral CRP may be significantly increased for CD patients after stroke, compared to those of non-CD patients. Subgroup analyses showed that CRP was higher in CD than that in non-CD patients when the mini-mental state examination was used. A higher level of CRP in the acute phase of ischemic stroke may suggest an increased risk of CD after stroke. However, these results should be cautiously interpreted because of the limited sample sizes and the diversity of potential confounders in the studies included in this meta-analysis.

## 1. Background

Cognitive decline (CD) after stroke is a common complication after stroke [1], encompassing a series of syndromes from mild cognitive impairment to dementia caused by stroke. One previous study reported that 57.7% of stroke patients exhibited varying degrees of cognitive decline at 3–6 months [2], and other studies suggested that 7.4–41.3% of patients met the criteria for dementia one year after stroke [3]. The presence of CD increases the risk of disability, recurrent stroke [4], and mortality [5], causes substantial pain for patients and difficulty for medical workers [6] and imposes a severe burden on the health care system and society. Therefore, the extent, impact, and long-term sequelae of CD underscore the need for assessment, treatment, and prognosis for patients.

Cognitive decline after stroke is typically treated with cholinesterase inhibitors and memantine, which are also commonly prescribed to people with Alzheimer’s dementia. Few studies have examined the effectiveness of these medications in stroke survivors as there is insufficient evidence to recommend the use of cholinesterase inhibitors, memantine nootropics, or cognitive rehabilitation for CD patients [7]. Therefore, we need to prevent and diagnose CD after stroke as early as possible, so that we can intervene and treat it early. Hence, identifying the risk factors of CD is crucial. In recent years, several studies have indicated that the discovery of differential concentrations of molecules in the blood serum and plasma of CD patients at the early stages of stroke can be helpful for elucidating CD pathogenesis. Such molecules may play essential roles in the diagnosis and prediction of CD after stroke.

C-reactive protein (CRP) is a protein synthesized by the liver. It can be measured using a more sensitive method called high-sensitivity C-reactive protein (Hs-CRP), which can detect lower levels of CRP [8]. CRP serves as a biomarker for general inflammation [9], and emerging evidence indicates that changes in CRP expression may be associated with cognitive decline in neurodegenerative diseases [10]. Meanwhile, the systemic inflammatory response to acute stroke involves increased CRP [11], which may be associated with the development of cognitive decline and dementia [12]. The relationship between C-reactive protein (CRP) and cognitive decline following a stroke remains controversial and is currently a subject of active research. This association is not yet well-defined, leading to an ongoing exploration within the research community to determine the role of CRP in cognitive decline after stroke. Fang Ran et al. and Chen Zhu, in 2019 [13,14], suggested that levels of Hs-CRP were significantly different between CD and non-CD (non-cognitive decline after stroke) patients. However, Mao et al. [15] suggested that there was no significant difference in Hs-CRP content between CD and non-CD patients. The controversy surrounding the relationship between CRP levels and cognitive decline after stroke can be attributed to several factors. Methodological differences among studies, variations in study populations, and the complex underlying mechanisms linking CRP to cognitive decline all contribute to the inconsistent findings. Further research is needed to clarify this relationship and better understand the role of CRP in cognitive decline after stroke.

Hence, the aim of the current study was to investigate whether or not CRP in the acute stage of stroke is associated with the development of CD. Meta-analyses were used to longitudinally compare the baseline concentration of CRP in the blood of acute stroke patients with cognitive decline and without cognitive decline. Furthermore, subgroup analysis and sensitivity analysis were also performed based on potential confounders to distinguish the source of between-study variation.

## 2. Methods

### 2.1. Search Strategy

The meta-analysis was performed according to the PRISMA (Preferred Reporting Items for Systematic Reviews and Meta-Analyses) guidelines [16]. We searched PubMed, Embase, Web of Science, and the Cochrane Library for articles published in English no later than 6 March 2023, using the following keywords: “(CRP OR C-reactive protein OR hsCRP OR hs-CRP or cytokines) AND (cerebrovascular disorders OR stroke) AND (cognition disorders OR cognition decline)” Details of the search strategies are shown in Supplementary Content S1. We also searched the reference lists of included studies and related reviews about CD after stroke.

### 2.2. Selection Criteria

Inclusion criteria for this meta-analysis were the following: (1) longitudinal studies, including case–control studies and cohort studies; (2) patients who were diagnosed with acute ischemic stroke verified via computed tomography or magnetic resonance imaging; (3) the use of scales to assess patients’ cognitive function; (4) measured CRP concentrations in peripheral blood in the acute stage of ischemic stroke (within two weeks after stroke). Exclusion criteria included the following: (1) data that could not be extracted; (2) reviews, case reports, conference summaries, protocols, and dissertations; (3) duplicate studies; and (4) studies in any language other than English. Two researchers independently screened and selected the studies, and any disagreements were supervised by the third researcher.

### 2.3. Data Extraction and Quality Assessment

We constructed Excel spreadsheets to extract data from the included articles. The main items included the first author, published year, country, research type, sample size, number of male and female participants, sample collection time, sample type, type of cognitive assessment scales, sample collection time, cognitive evaluation time, and source of CRP. For CRP concentrations, mean values, standard deviations (SDs), and numbers of participants for each group were extracted. When the concentrations of CRP levels were reported in other formats (e.g., medians, standard errors, confidence intervals, interquartile ranges, or *p* values), a standard method was used to estimate the mean value and SDs [17,18]. For studies in which CRP levels were measured at multiple time points, only the data from the first test was used.

The Newcastle–Ottawa scale (NOS) was used to evaluate the quality of the included studies with a total score of nine stars. For cohort studies, the NOS assesses three components: selection, comparability, and outcomes [19]. Each item could be given one star at most, while a maximum of two stars could be given for comparability. Studies were defined as high (>7 stars), medium (6–7 stars), or low quality (<6 stars).

Data extraction and quality assessment were conducted independently by two researchers, and any differences were resolved by consulting a third researcher.

### 2.4. Data Analysis

RevMan5.4 software (Cochrane Informatics and Knowledge Management Department, London, UK) and Stata version 14.0 (Stata Corp, LP, USA, College Station, TX, USA) were used for the analysis of the data. Meta-analyses comparing peripheral blood CRP concentrations between CD and non-CD patients were performed. Because of the different detection methods between studies, we utilized the standard mean difference (SMD) as the effect size for the differences in CRP levels and calculated corresponding 95% confidence intervals (CIs). Considering the underlying heterogeneity among studies, we used random-effect models for all meta-analyses, with a *p* value of <0.05 being considered statistically significant. Cochrane’s Q test was used to assess heterogeneity among studies, measuring the degree of deviation between the SMD of a single study and combined studies, where *p* < 0.1 was considered to be significant and considered to indicate moderate heterogeneity, and over 75% was regarded as high heterogeneity [20]. An optimistic pooled SMD estimate indicates increased CRP levels in CD patients compared with those in non-CD patients. For evaluating possible publication bias, both a funnel plot and Egger regression asymmetry test were performed [21]. Meta-regression analysis was performed based on countries, cognitive evaluation time, the proportion of males, quality of articles, age, proportion of diabetes cases, proportion of hypertension cases, proportion of hyperlipidemia cases, proportion of smokers, proportion of people who drink, proportion of recurrent stroke cases and scores from the National Institutes of Health Stroke Scale. In addition, to investigate the stability of the meta-analytic results, pooled effect sizes were recalculated after a single study was removed in turn. The null hypotheses of each included study and the meta-analysis were tested with a 0.05 α error [22]. Considering the differences in units between studies, SMDs were used when evaluating the statistical power of the meta-analysis [23].

## 3. Results

### 3.1. Study Selection

**Figure 1:**
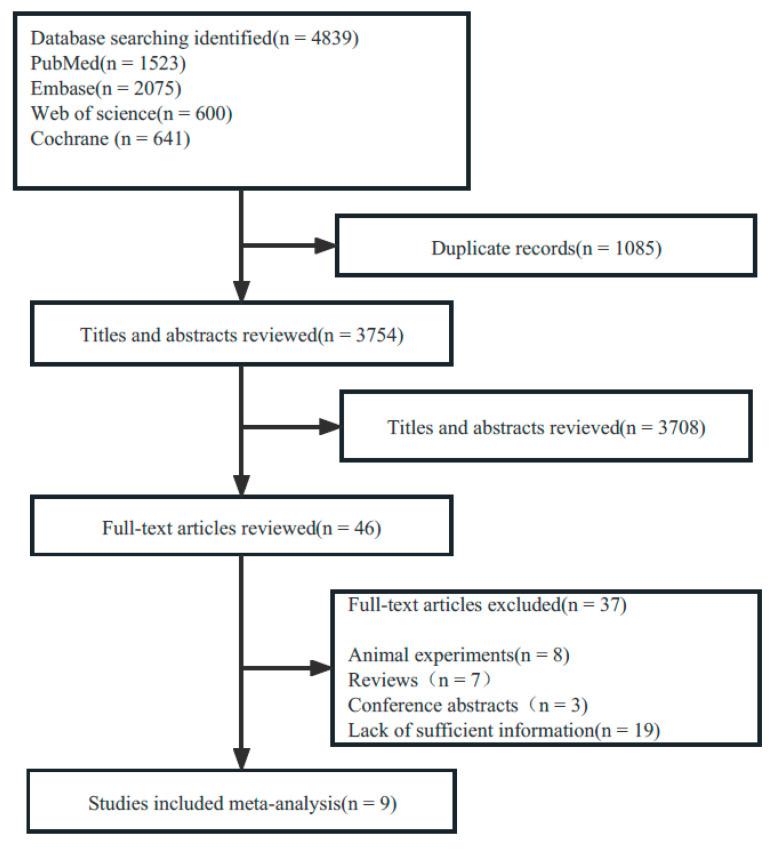
PRISMA flow diagram.

A total of 4839 publications were retrieved up to 6 March 2023. After removing duplicates, 3754 publications were retained for the title and abstract screening. Full texts from 46 publications were reviewed according to the inclusion and exclusion criteria. Nine studies reporting animal experiments and seven reviews were excluded. We also excluded 3 conference abstracts and 19 articles containing insufficient information. Consequently, nine original studies were included. The flowchart of the study selection is shown in Figure 1.

### 3.2. Characteristics of Included Studies and Quality Assessments

The characteristics of included studies are shown in Table 1. In total, 3893 patients were included in this meta-analysis. Of these, 1695 participants developed CD, and 2198 did not. The reported incidence of CD ranged from 29% to 65%. The participants consisted of 2254 men and 1639 women (58% men). The included studies were published between 2013 and 2022, with sample sizes ranging from 31 to 1116. CRP samples were derived from the serum (eight studies) and plasma (one study) of stroke patients in the acute phase. Moreover, the overall quality of the studies included in this meta-analysis was moderate to high, and the quality analysis assessment via the NOS (Supplementary Content S2) revealed that all of the studies achieved ratings of more than six stars, with two papers rated as seven stars [24,25], six rated as eight stars [13,14,15,26,27,28], and one rated as nine stars [29].

### 3.3. CRP Level at Baseline of Stroke and Cognitive Impairment

#### 3.3.1. Meta-Analysis

**Figure 2:**
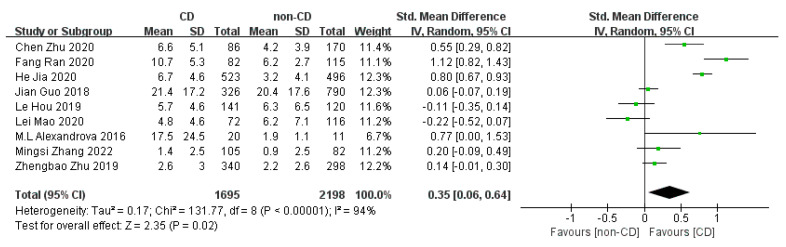
Forest plot of CRP levels in CD and non-CD patients [13,14,15,24,25,26,27,28,29].

Nine studies comparing peripheral CRP concentrations between CD and non-CD patients (1695 CD patients and 2198 non-CD patients) were used for the meta-analyses. The pooled SMD estimated via random effect models showed a significant increase in CRP levels in CD patients versus non-CD patients (SMD = 0.35, 95% CI = (0.06; 0.64); *p* = 0.020), with high heterogeneity (I^2^ = 94%, *p* < 0.001) (Figure 2).

#### 3.3.2. Meta-Regression

In the meta-regression analysis of comparing CRP levels between CD and non-CD patients, we failed to find evidence of statistical correlations between CRP concentrations and countries (*p* = 0.760), as well as the cognitive evaluation time (*p* = 0.287), proportion of males (*p* = 0.310), quality of articles (*p* = 0.173), age (*p* = 0.330), the proportion of diabetes patients (*p* = 0.542), proportion of hypertension patients (*p* = 0.315), proportion of hyperlipidemia patients (*p* = 0.919), proportion of smokers (*p* = 0.816), proportion of patients who drink (*p* = 0.203), proportion of recurrent stroke patients (*p* = 0.667) and scores from the National Institutes of Health Stroke Scale (*p* = 0.300).

#### 3.3.3. Subgroup Analysis

We performed five subgroup analyses, and the results of subdividing groups based on the clinical and methodological differences of studies were collected and presented in Table 2. Forest maps are shown in Supplementary Content S3. In most subdividing group analyses that included more than one study, the conclusion results suggested that patients with CD had higher CRP levels during the acute stroke period than did patients without cognitive impairment symptoms. In the subgroup analysis stratified based on the scales for cognitive assessment, patients with CD had higher CRP levels than did patients without CD when the MMSE (SMD = 0.54; 95%CI = (0.13; 0.94); *p* = 0.01) was used, while no significant difference in CRP between groups was observed if the cognitive function was assessed using other scales (SMD = 0.20; 95%CI = (−0.18; 0.58); *p* = 0.40), so the overall subgroup difference was not statistically significant (*p* = 0.24). The stratification of the studies based on the detection sensitivity of CRP revealed that there as a significant difference in either CRP levels or Hs-CRP levels between patients with CD and the controls; the overall subgroup difference was not statistically significant (*p* = 0.97). Stratification analysis of the studies categorized according to the study design (prospective study and retrospective study) showed that CRP levels were significantly higher in cases than in controls in both subgroups, so the difference in CRP levels between the prospective study and retrospective study was significant (*p*  =  0.0003). Stratification of the studies based on the sample size (N < 500 and N ≥ 500) showed that there was no significant difference in CRP levels between patients with CD and patients without CD in either subgroup, so the overall subgroup difference was not statistically significant (*p*  =  0.94). Stratification of the studies based on the source of CRP (serum and plasma) showed that the CRP level was significantly higher in cases than in controls in both subgroups; the overall subgroup difference was not statistically significant (*p*  =  0.28).

### 3.4. Publication Bias

**Figure 3:**
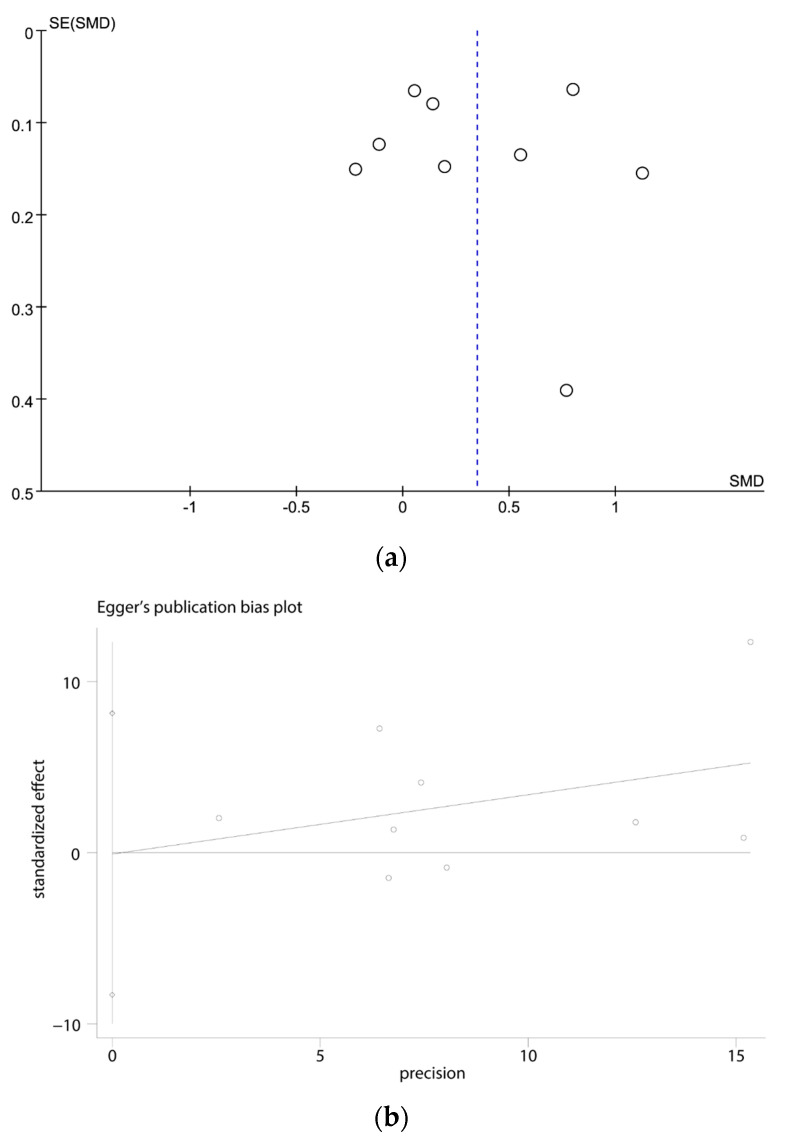
(**a**) Funnel plots of the meta-analysis; (**b**) Egger’s test for the publication bias.

The funnel plot (Figure 3a) and Egger’s test (Figure 3b) indicated that there was no potential risk of publication bias (t = −0.29, *p* = 0.782).

## 4. Discussion

To our knowledge, this is the first meta-analysis investigating the relationship between acute ischemic stroke-related CRP levels in the blood and subsequent cognitive decline after stroke. Nine cohort studies with 3893 stroke patients were analyzed, which revealed that patients with higher CRP levels in the acute stage of ischemic stroke might have higher risks of CD (Figure 2).

CD is a common complication after ischemic stroke, but the exact incidence has yet to be uniformly stated. We included a total of nine articles with incidence rates ranging from 29% to 65%, with a mean incidence of 44%, and this result was in accord with the findings of previous studies [2,30,31]. It is suggested that CD is a common post-stroke complication and requires to be considered high-priority.

The relationship between stroke and cognitive decline is intricate due to a variety of factors at play [32]. Lei Zhao [33] explored the different impacts on cognitive function based on the different infarct locations through their research. Additionally, Ryan S Falck [34] found that sleep can also influence cognitive function after stroke. Moreover, the presence of other vascular risk factors and the overall health status of individuals have been found to be associated with cognitive decline after stroke [35]. This intricate relationship necessitates a comprehensive understanding of the underlying mechanisms responsible for cognitive decline after a stroke. This understanding is essential for the development of preventive or management strategies aimed at addressing this complication.

One potential contributor to cognitive decline following a stroke is inflammation, marked by various biological processes including elevated C-reactive protein (CRP) levels [36]. Since the diagnosis of cognitive decline currently hinges on various scales [37], medical professionals are increasingly interested in exploring biological markers such as CRP for early detection. Consequently, the current efforts of clinicians and scientists are oriented towards identifying such markers to augment the diagnosis of cognitive decline following stroke.

The primary analysis in our meta-analysis of nine studies revealed that, compared with non-CD patients, concentrations of CRP in the acute stage of stroke were higher in patients with CD than those in patients without CD. Furthermore, the current studies suggest that CRP contributes to cognitive impairment at least through two mechanisms [38,39]. Firstly, CRP may induce cerebral atherosclerosis and trigger microvascular and macrovascular lesions by mediating the uptake of low-density lipoprotein by macrophages and promoting foam cell formation and by impounding endothelial function or by inducing the abnormal migration and proliferation of human vascular smooth muscle cells. Brain damage disrupts the integrity of the prefrontal–subcortical circuit, which leads to cognitive decline [39,40]. Secondly, CRP may damage brain tissue by activating the classical complement system, which contributes to cognitive function. This result was consistent with the findings of previous meta-analyses. In an analysis of 5255 non-demented subjects from four prospective studies, Yang Jin et al. [10] found a weak association between peripheral CRP levels and cognitive decline in subjects without dementia. Kuo Hsu-Ko et al. [41] pooled data from healthy adults and reported that high concentrations of CRP are associated with an increased risk of stroke and cognitive impairment in healthy adults.

Additionally, subgroup analysis revealed that when the cognitive assessment was conducted using the MMSE, the baseline blood CRP concentrations in CD patients were higher than those in non-CD patients. This difference disappeared when patients were assessed using other scales. Although Zhu Yueli et al. [42] found that both the MMSE and the MoCA were good screening instruments for cognitive impairment during the acute phase in stroke patients, but the accuracy of the MoCA in 3 months and 6–12 months after stroke remains to be studied. The MMSE was higher in specificity than the MoCA and is the most widely used post-stroke cognitive function scale internationally [43]. Therefore, in the current study, we analyzed patients assessed with the MMSE as a separate subgroup. Because some previous studies reported that the MMSE was lacking in sensitivity after acute stroke, the degree of cognitive impairment in patients with post-stroke cognitive dysfunction measured via the MMSE may be slightly more substantial than that measured using other scales [44,45,46]. This finding suggests that CRP may be more closely related to moderate-to-severe post-stroke cognitive dysfunction. However, the literature on this topic is limited, and this possibility should be further explored. The relationship between CRP and different degrees of cognitive impairment after stroke should be explored in future research.

Hs-CRP is a more sensitive measurement method for CRP that can detect lower levels of CRP. Although our subgroup analysis suggests there is not a significant correlation between Hs-CRP and cognitive decline after stroke, there are studies indicating a relationship between Hs-CRP and non-vascular cognitive functions. Research conducted by Gelin Xu [47] et al. demonstrated that enhanced serum Hs-CRP levels are associated with cognitive deterioration in the elderly without a history of cerebrovascular events. Additionally, studies from Guangyao Wang [48] and his team suggest a connection between Hs-CRP and poor outcomes one year after stroke. Therefore, future research could further explore the relationship between Hs-CRP and cognitive decline after stroke.

Our study encompasses several limitations. Initially, we observed heterogeneity in the samples involved in this meta-analysis, attributable in part to the inclusion of studies with limited sample sizes, variable designs, and methodologies. Furthermore, the lack of information in our selected literature regarding potential influential factors such as age or stroke severity may have introduced bias. While the majority of included studies excluded patients with pre-existing cognitive decline and those with other inflammatory diseases, the criteria for inclusion and exclusion still varied across the board. This variation likely contributed to the heterogeneity we observed in our results. Secondly, the majority of the literature incorporated in this study assessed cognitive function within six months or less. The conclusions derived from these short-term studies may differ from those that could be drawn from long-term research. Thirdly, stroke could potentially accelerate the development of other forms of cognitive impairment. Hence, our conclusions are not specific to vascular cognitive decline, warranting further exploration in future studies. Lastly, we identified that different detection methods between CRP and Hs-CRP, although not the primary source of heterogeneity in our study, may still influence the results to a degree. This could introduce unaccounted variability in our current model. We therefore recognize this as a potential limitation and advocate for future studies to either standardize these methods or account for their influence to assure more consistent and reliable outcomes. In order to address these limitations, it is necessary to conduct additional well-designed studies that incorporate larger sample sizes, standardized methodologies, extended follow-up periods, and a meticulous control of confounding factors.

## 5. Conclusions

Through a meta-analysis of 3893 subjects, we observed that peripheral CRP levels could be significantly elevated in patients with cognitive decline (CD) compared to those in patients without CD. This suggests a potential link between increased CRP levels and cognitive decline post-stroke. However, while meta-analyses can consolidate existing evidence, their conclusions must be considered cautiously due to the inherent limitations, such as heterogeneity across studies and possible publication bias. Given the evolving nature of research in this field, it is crucial to continuously incorporate new findings and advancements in our understanding.

## Figures and Tables

**Table 1:** Characteristics of studies included.

					CD	Non-CD			CRP Level (Mean ± SD, mg/dL)		Hs-CRP Level (Mean ± SD, mg/dL)						
Author	Year	Ref.	Country	Research Type	Numbers (n)	Numbers (n)	Total Numbers (n)	Male Sex, n (%)	CD	Non-CD	CD	Non-CD	Cognitive Evaluation Time	Scale	Source of CRP	Sample Collection Time	NOS
Chen Zhu	2020	[14]	China	prospective study	86	170	256	139 (54)	/	/	6.6 ± 5.1	4.2 ± 3.9	after 1 year	MMSE	plasma	within 24 h after admission	8
Fang Ran	2020	[13]	China	prospective study	82	115	197	84 (43)			10.7 ± 5.3	6.2 ± 2.7	NA	MoCA	serum	at admission	8
He Jia	2020	[24]	China	retrospective study	523	496	1019	531 (52)	6.7 ± 4.6	3.2 ± 4.1	/	/	3 months poststroke	MMSE	serum	within 24 h of admission	7
Jian Guo	2018	[29]	China	prospective study	326	790	1116	631 (57)	21.4 ± 17.2	20.4 ± 17.6	/	/	6 months after stroke	the Six-Item Screener	Serum	within one week of stroke onset	9
Le Hou	2019	[26]	China	prospective study	141	120	261	140 (54)	/	/	5.7 ± 4.6	6.3 ± 6.5	3 months After the stroke	MoCA	serum	in the morning after admission	8
Lei Mao	2020	[15]	China	prospective study	72	116	188	117 (62)	/	/	4.8 ± 4.6	6.2 ± 7.1	1 year after stroke	MoCA	serum	within 24 h of admission	8
M.L Alexandrova	2016	[25]	Bulgaria	prospective study	20	11	31	16 (52)	/	/	17.5 ± 24.5	1.9 ± 1.1	12 months After the stroke	MMSE	serum	at admission	7
Mingsi Zhang	2022	[28]	China	prospective study	105	82	187	148 (79)	1.37 ± 2.48	0.9 ± 2.5	/	/	within 2 weeks	MoCA	serum	at admission	8
Zhengbao Zhu	2019	[27]	China	prospective study	340	298	638	448 (70)	/	/	2.6 ± 3.0	2.2 ± 2.6	3 months after acute ischemic stroke	MMSE	serum	within 24 h of hospital admission	8

Abbreviations: MMSE, mini-mental state examination; MoCA, Montreal Cognitive Assessment.

**Table 2:** Results of subgroup analyses.

	Studies	Comparison Statistics				Heterogeneity				*p*-Value between Subgroups
		SMD	95% CI	Z	*p*-Value	Q	df	*p*-Value	I2 (%)	
CD VS non-CD										
Scales for cognitive assessment										
MMSE	4	0.54	0.13, 0.94	2.59	0.01	41.58	3	<0.01	93	0.24
Other kinds of scales	5	0.20	−0.18, 0.58	1.05	0.29	51.39	4	<0.01	92	
Detection sensitivity of CRP										
CRP	3	0.36	−0.19, 0.90	1.29	0.20	66.86	2	<0.01	97	0.97
Hs-CRP	6	0.34	−0.04, 0.72	1.77	0.08	58.27	5	<0.01	91	
Research types										
Prospective study	8	0.27	0.02, 0.53	2.11	0.03	62.88	7	<0.01	89	/
Retrospective study	1	0.80	0.67, 0.93	12.30	/	/	/	/	/	
Sample size										
N < 500	6	0.36	−0.07, 0.79	1.66	0.10	56.22	5	<0.01	91	0.94
N ≥ 500	3	0.33	−0.15, 0.82	1.36	0.17	74.62	2	<0.01	97	
Source of CRP										
serum	8	0.32	0.00, 0.64	1.98	0.05	129.09	7	<0.01	95	/
plasma	1	0.55	0.29, 0.82	4.10	/	/	/	/	/	

Abbreviations: CI, confidence interval; df, degrees of freedom; SMD, standardized mean difference.

## Data Availability

No new data were created or analyzed in this study. Data sharing is not applicable to this article.

## Supplementary Materials

The following supporting information can be downloaded at https://www.mdpi.com/article/10.3390/brainsci13071082/s1. Supplementary Content S1: Search strategy and results; Supplementary Content S2: Methodological quality of cohort studies included in the meta-analysis; Supplementary Content S3: Forest maps of subgroups.
