# Supplementary material for: C-Reactive Protein Levels and Cognitive Decline following Acute Ischemic Stroke: A Systematic Review and Meta-Analysis
Source: Brain Sci. 2023 Jul 17;13(7):1082. doi: 10.3390/brainsci13071082 (PMC10377587; doi:10.3390/brainsci13071082)

C-reactive protein levels and cognitive decline after acute ischemic stroke: An Systematic Review and Meta-analysis Supplementary Content to the manuscript

Contents of Supplementary Content

Supplementary Content S1	Search strategy and results
Supplementary Content S2	Methodological quality of cohort studies included in the meta-analysis
Supplementary Content S3	Forest maps of subgroups

Supplementary Content S1

Search strategy and results

Number of citations by each database

**Databases and Trial registers:**	**Citations**
**Databases:**	
PubMed	1523
Cochrane	641
Embase	2075
Web of Science	600
**Total (databases)**	**4839**

Full search strategy for each database

PubMed

#1 ((Cerebrovascular Disorders [MeSH Major Topic]) OR (Hemiplegia [MeSH Major Topic])) OR (Paresis[MeSH Major Topic])

#2 hemipleg*[Title] OR hemipar*[Title] OR paresis[Title] OR paretic[Title] OR stroke[Title] OR poststroke[Title] OR post?stroke[Title] OR cerebrovasc*[Title] OR brain vasc*[Title] OR cerebral vasc*[Title] OR cva*[Title] OR apoplex*[Title] OR SAH[Title]

#3 ((brain*[Title] OR cerebr*[Title] OR cerebell*[Title] OR intracran*[Title] OR intracerebral) [Title]) AND ((isch*emi*[Title] OR infarct*[Title] OR thrombo*[Title] OR emboli*[Title] OR occlus*))[Title])

#4 ((brain*[Title] OR cerebr*[Title] OR cerebell*[Title] OR intracerebral [Title] OR intracranial [Title] OR subarachnoid) [Title]) AND ((haemorrhage*[Title] OR hemorrhage*[Title] OR haematoma*[Title] OR hematoma*[Title] OR bleed*) [Title])

#5 (((#1) OR (#2)) OR (#3)) OR (#4)

#6 (Dementia [MeSH Major Topic]) OR (Cognition Disorders [MeSH Major Topic])

#7 Agnosia [Title/Abstract] OR amnesia [Title/Abstract] OR confusion [Title/Abstract] OR inattention [Title/Abstract] OR Dementia [Title/Abstract]

#8 ((cogniti*[Title/Abstract] OR arous*[Title/Abstract] OR orientat*[Title/Abstract] OR attention*[Title/Abstract] OR concentrat*[Title/Abstract] OR memor*[Title/Abstract] OR recall[Title/Abstract] OR percept*[Title/Abstract] OR think*[Title/Abstract] OR sequenc*[Title/Abstract] OR algorithm*[Title/Abstract] OR judg?ment*[Title/Abstract]

OR awareness[Title/Abstract] OR problem solving[Title/Abstract] OR generali?ation[Title/Abstract] OR transfer[Title/Abstract] OR comprehension[Title/Abstract] OR learning)[Title/Abstract] AND (disorder*[Title/Abstract] OR declin*[Title/Abstract] OR dysfunct*[Title/Abstract] OR impair*[Title/Abstract] OR deficit*[Title/Abstract] OR abilit*[Title/Abstract] OR problem*))[Title/Abstract]

#9 dysexecutive syndrome*[Title/Abstract] OR mental process* [Title/Abstract] OR impulsive behavio?r*[Title/Abstract] OR executive function*[Title/Abstract]

#10 (((#6) OR (#7)) OR (#8)) OR (#9)

#11 Psd [Title/Abstract] OR psci [Title/Abstract]

#12 (#10) AND (#5)

#13 (#11) OR (#12)

#14 ((Cytokines [MeSH Major Topic]) OR (C-reactive protein [MeSH Major Topic]))

#15 Cytokine* OR Inflammat* OR Pro-inflammat* or CRP or C-reactive protein or hs-CRP or hsCRP

#16 (#14) OR (#15)

#17 ((#13) AND (#16))

#18 plasma or blood or serum

#19 ((#17) AND (#18))

Cochrane

#1 MeSH descriptor: [Cerebrovascular Disorders] explode all trees

#2 MeSH descriptor: [Hemiplegia] explode all trees

#3 MeSH descriptor: [Paresis] explode all trees

#4 (hemipleg* or hemipar* or paresis or paretic or stroke or poststroke or post-stroke or cerebrovasc* or brain vasc* or cerebral vasc* or cva* or apoplex* or SAH): ti

#5 (hemipleg* or hemipar* or paresis or paretic or stroke or poststroke or post-stroke or cerebrovasc* or brain vasc* or cerebral vasc* or cva* or apoplex* or SAH): ab

#6 ((brain* or cerebr* or cerebell* or intracerebral or intracranial or subarachnoid) near/5 (haemorrhage* or hemorrhage* or haematoma* or hematoma* or bleed*)): ti

#7 ((brain* or cerebr* or cerebell* or intracerebral or intracranial or subarachnoid) near/5 (haemorrhage* or hemorrhage* or haematoma* or hematoma* or bleed*)): ab

#8 (((brain* or cerebr* or cerebell* or intracran* or intracerebral) near/5 (isch*emi* or infarct* or thrombo* or emboli* or occlus*))): ti

#9 (((brain* or cerebr* or cerebell* or intracran* or intracerebral) near/5 (isch*emi* or infarct* or thrombo* or emboli* or occlus*))): ab

#10 (((cogniti* or arous* or orientat* or attention* or concentrat* or memor* or recall or percept* or think* or sequenc* or algorithm* or judg?ment* or awareness or problem solving or generali?ation or transfer or comprehension or learning) near/10 (disorder* or declin* or dysfunct* or impair* or deficit* or abilit* or problem*))):ti OR (((cogniti* or arous* or orientat* or attention* or concentrat* or memor* or recall or percept* or think* or sequenc* or algorithm* or judg?ment* or awareness or problem solving or generali?ation or transfer or comprehension or learning) near/10 (disorder* or declin* or dysfunct* or impair* or deficit* or abilit* or problem*))):ab

#11 ((dysexecutive syndrome* or mental process* or impulsive behavio?r* or executive

function*)):ti OR ((dysexecutive syndrome* or mental process* or impulsive behavio?r* or executive function*)):ab

#12 (Psd or psci): ti OR (Psd or psci): ab

#13 MeSH descriptor: [Cytokines] explode all trees

#14 MeSH descriptor: [C-reactive protein] explode all trees

#15 (CRP or C-reactive protein or hs-CRP or hsCRP): ti OR (CRP or C-reactive protein or hs-CRP or hsCRP): ab

#16 #1 OR #2 OR #3 OR #4 OR #5 OR #6

#17 10 OR #11

#18 #16 AND #17

#19 #18 OR #12

#20 #13 OR #14 OR #15

#21 #18 AND #20

#22 (plasma or blood or serum): ti AND (plasma or blood or serum): ab

#23 #21 AND #22

Embase

#1 'cerebrovascular disease'/exp OR 'hemiplegia'/exp OR 'paresis'/exp

#2 hemipleg*: ti OR hemipar*: ti OR paresis: ti OR paretic: ti OR stroke: ti OR poststroke: ti OR post-stroke: ti OR cerebrovasc*: ti OR' brain vasc*': ti OR' cerebral vasc*': ti OR cva*: ti OR apoplex*: ti OR sah: ti

#3 'cognitive defect'/exp OR' dementia'/exp

#4 agnosia: ti OR amnesia: ti OR confusion: ti OR inattention: ti OR dementia: ti

#5 psd: ti OR psci: ti

#6 'cytokine'/exp OR' creactive protein'/exp

#7 cytokine*: ab,ti OR inflammat*: ab,ti OR' pro inflammat*': ab,ti OR' tumor necrosis factor-alpha': ab,ti OR' tumor necrosis factor-α': ab,ti OR' tnf alpha': ab,ti OR' tnf α': ab,ti OR interleukin*: ab,ti OR' il 1*': ab,ti OR' il 2*': ab,ti OR' il 3*': ab,ti OR' il 4': ab,ti OR' il 5': ab,ti OR' il 6': ab,ti OR' il 7': ab,ti OR' il 8': ab,ti OR' il 9': ab,ti OR crp: ab,ti OR' c-reactive protein': ab,ti OR' hs crp': ab,ti OR hscrp: ab,ti

#8 plasma: ab,ti OR blood: ab,ti OR serum: ab,ti

#9 #1 OR #2

#10 #3 OR #4

#11 #9 AND #10

#12 #5 OR #11

#13 #6 OR #7

#14 #8 AND #12 AND #13

Web of Science

#1 TI=(Cerebrovascular Disorders or Hemiplegia or Paresis or hemipleg* or hemipar* or paresis or paretic or stroke or poststroke or post-stroke or cerebrovasc* or brain vasc* or cerebral vasc* or cva* or apoplex* or SAH）

#2 TI=((brain* or cerebr* or cerebell* or intracran* or intracerebral) and (isch*emi* or infarct* or thrombo* or emboli* or occlus*)) OR TI=（(brain* or cerebr* or cerebell* or intracerebral or intracranial or subarachnoid) and (haemorrhage* or hemorrhage* or haematoma* or hematoma* or bleed*))

#3 TI = (Cognition Disorders or Dementia) OR AB = (Cognition Disorders or Dementia)

#4 TI = (agnosia or amnesia or confusion or inattention or Dementia) OR AB = (agnosia or amnesia or confusion or inattention or Dementia)

#5 TI=((cogniti* or arous* or orientat* or attention* or concentrat* or memor* or recall or percept* or think* or sequenc* or algorithm* or judg?ment* or awareness or problem solving or generali?ation or transfer or comprehension or learning) AND (disorder* or declin* or dysfunct* or impair* or deficit* or abilit* or problem*)) OR AB=((cogniti* or arous* or orientat* or attention* r concentrat* or memor* or recall or percept* or think* or sequenc* or algorithm* or judg?ment* or awareness or problem solving or generali?ation or transfer or comprehension or learning) AND (disorder* or declin* or dysfunct* or impair* or deficit* or abilit* or problem*))

#6 TI = (dysexecutive syndrome* or mental process* or impulsive behavio?r* or executive function*) OR AB=(dysexecutive syndrome* or mental process* or impulsive behavio?r* or executive function*)

#7 TI = (Psd or psci) OR AB = (Psd or psci)

#8 AB = (Cytokines or C-reactive protein) OR TI = (Cytokines or C-reactive protein)

#9 TI = (Cytokine* OR Inflammat* OR Pro-inflammat* OR Tumor Necrosis Factor-alpha OR Tumor Necrosis Factor-α OR TNF-alpha OR TNF-αOR interleukin* OR il-1* OR il-2* OR il-3* OR il-4 OR il-5 OR il-6 OR il-7 OR il-8 OR il-9 OR CRP or C-reactive protein or hs-CRP or hsCRP) OR AB = (Cytokine* OR Inflammat* OR Pro-inflammat* OR Tumor Necrosis Factor-alpha OR Tumor Necrosis Factor-α OR TNF-alpha OR TNF-αOR interleukin* OR il-1* OR il-2* OR il-3* OR il-4 OR il-5 OR il-6 OR il-7 OR il-8 OR il-9

OR CRP or C-reactive protein or hs-CRP or hsCRP)

#10 TI = (plasma or blood or serum) OR AB = (plasm or blood or serum)

#11 #2 OR #1

#12 #6 OR #5 OR #4 OR #3

#13 #12 AND #11

#14 #13 OR #7

#15 #9 OR #8

#16 #15 AND #14 AND #10

Supplementary Content S2

Methodological quality of cohort studies included in the meta-analysis

Study ID	Representative of the exposed group	Selection of the unexposed group	Confirm the exposure	Outcome of interest not present at the start of study	Control for important factor or additional factor	Outcome assessment	Follow-up long enough for outcomes to occur	Adequacy of follow up of cohorts4	Total quality scores
Chen Zhu 2020	☆	☆	☆	☆	☆☆	☆	☆	-	8
Fang Ran 2020	☆	☆	☆	☆	☆☆	☆	-	☆	8
He Jia 2020	☆	☆	☆	☆	☆☆	☆	-	-	7
Jian Guo 2018	☆	☆	☆	☆	☆☆	☆	☆	☆	9
Le Hou 2019	☆	☆	☆	☆	☆☆	☆	-	☆	8
Lei Mao 2020	☆	☆	☆	☆	☆☆	☆	☆	-	8
M.L Alexandrova 2016	☆	☆	☆	-	☆☆	☆	☆	-	7
Mingsi Zhang 2022	☆	☆	☆	☆	☆☆	☆	☆	-	8
ZhengbaoZhu 2019	☆	☆	☆	☆	☆☆	☆	-	☆	8

Supplementary Content S3

Forest maps of subgroups

Forest map of scales for cognitive assessment

Study or Subgroup	CD Mean	CD SD	CD Total	non-CD Mean	non-CD SD	non-CD Total	Weight	Std. Mean Difference IV, Random, 95% CI
**1.2.1 MMSE**								
Zhengbao Zhu 2019	2.6	3	340	2.2	2.6	298	12.2%	0.14 [-0.01, 0.30]
M.L Alexandrova 2016	17.5	24.5	20	1.9	1.1	11	6.7%	0.77 [0.00, 1.53]
He Jia 2020	6.7	4.6	523	3.2	4.1	496	12.3%	0.80 [0.67, 0.93]
Chen Zhu 2020	6.6	5.1	86	4.2	3.9	170	11.4%	0.55 [0.29, 0.82]
**Subtotal (95% CI)**			**969**			**975**	**42.6%**	**0.54 [0.13, 0.94]**
Heterogeneity: Tau² = 0.14; Chi² = 41.58, df = 3 (P < 0.00001); I² = 93%								
Test for overall effect: Z = 2.59 (P = 0.010)								
**1.2.3 Other kinds of scales**								
Mingsi Zhang 2022	1.4	2.5	105	0.9	2.5	82	11.2%	0.20 [-0.09, 0.49]
Lei Mao 2020	4.8	4.6	72	6.2	7.1	116	11.2%	-0.22 [-0.52, 0.07]
Le Hou 2019	5.7	4.6	141	6.3	6.5	120	11.6%	-0.11 [-0.35, 0.14]
Jian Guo 2018	21.4	17.2	326	20.4	17.6	790	12.3%	0.06 [-0.07, 0.19]
Fang Ran 2020	10.7	5.3	82	6.2	2.7	115	11.1%	1.12 [0.82, 1.43]
**Subtotal (95% CI)**			**726**			**1223**	**57.4%**	**0.20 [-0.18, 0.58]**
Heterogeneity: Tau² = 0.17; Chi² = 51.39, df = 4 (P < 0.00001); I² = 92%								
Test for overall effect: Z = 1.05 (P = 0.29)								
**Total (95% CI)**			**1695**			**2198**	**100.0%**	**0.35 [0.06, 0.64]**
Heterogeneity: Tau² = 0.17; Chi² = 131.77, df = 8 (P < 0.00001); I² = 94%								
Test for overall effect: Z = 2.35 (P = 0.02)								
Test for subgroup differences: Chi² = 1.39, df = 1 (P = 0.24), I² = 28.1%								

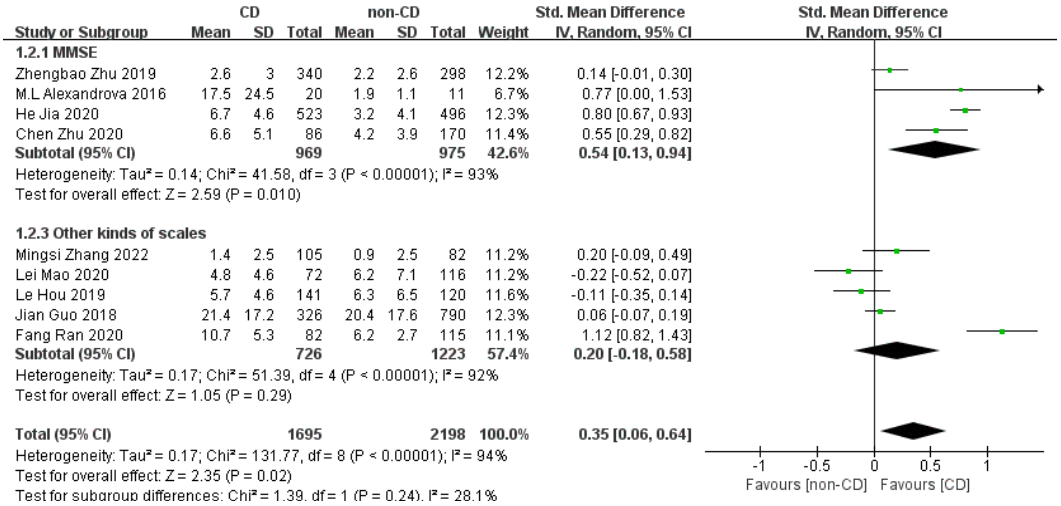

Forest map of detection sensitivity of CRP

Study or Subgroup	CD Mean	CD SD	CD Total	non-CD Mean	non-CD SD	non-CD Total	Weight	Std. Mean Difference IV, Random, 95% CI
**1.3.1 CRP**								
He Jia 2020	6.7	4.6	523	3.2	4.1	496	12.3%	0.80 [0.67, 0.93]
Jian Guo 2018	21.4	17.2	326	20.4	17.6	790	12.3%	0.06 [-0.07, 0.19]
Mingsi Zhang 2022	1.4	2.5	105	0.9	2.5	82	11.2%	0.20 [-0.09, 0.49]
**Subtotal (95% CI)**			**954**			**1368**	**35.8%**	**0.36 [-0.19, 0.90]**
Heterogeneity: Tau² = 0.22; Chi² = 66.86, df = 2 (P < 0.00001); I² = 97%								
Test for overall effect: Z = 1.29 (P = 0.20)								
**1.3.2 Hs-CRP**								
Chen Zhu 2020	6.6	5.1	86	4.2	3.9	170	11.4%	0.55 [0.29, 0.82]
Fang Ran 2020	10.7	5.3	82	6.2	2.7	115	11.1%	1.12 [0.82, 1.43]
Le Hou 2019	5.7	4.6	141	6.3	6.5	120	11.6%	-0.11 [-0.35, 0.14]
Lei Mao 2020	4.8	4.6	72	6.2	7.1	116	11.2%	-0.22 [-0.52, 0.07]
M.L Alexandrova 2016	17.5	24.5	20	1.9	1.1	11	6.7%	0.77 [0.00, 1.53]
Zhengbao Zhu 2019	2.6	3	340	2.2	2.6	298	12.2%	0.14 [-0.01, 0.30]
**Subtotal (95% CI)**			**741**			**830**	**64.2%**	**0.34 [-0.04, 0.72]**
Heterogeneity: Tau² = 0.19; Chi² = 58.27, df = 5 (P < 0.00001); I² = 91%								
Test for overall effect: Z = 1.77 (P = 0.08)								
**Total (95% CI)**			**1695**			**2198**	**100.0%**	**0.35 [0.06, 0.64]**
Heterogeneity: Tau² = 0.17; Chi² = 131.77, df = 8 (P < 0.00001); I² = 94%								
Test for overall effect: Z = 2.35 (P = 0.02)								
Test for subgroup differences: Chi² = 0.00, df = 1 (P = 0.97), I² = 0%								

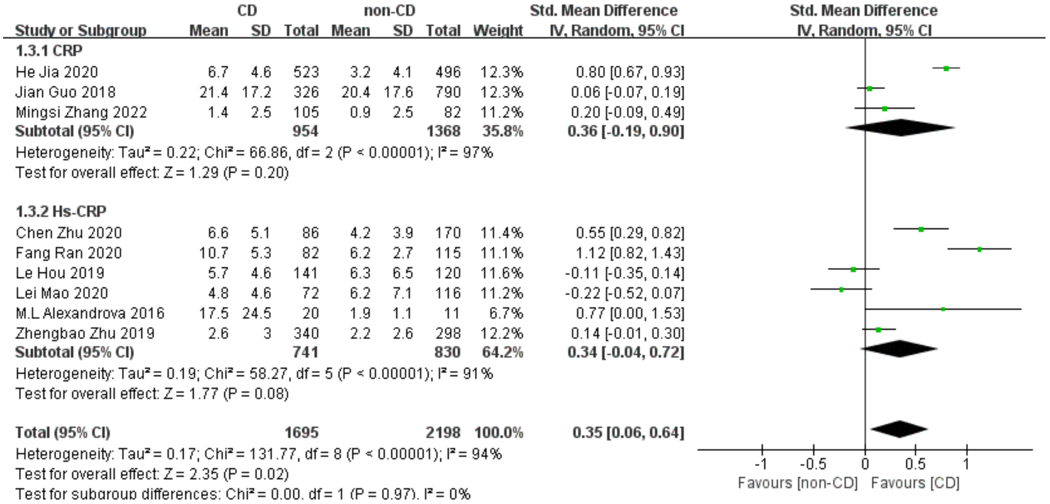

Forest map of research types

Study or Subgroup	CD Mean	CD SD	CD Total	non-CD Mean	non-CD SD	non-CD Total	Weight	Std. Mean Difference IV, Random, 95% CI
**1.4.1 Prospective study**								
Chen Zhu 2020	6.6	5.1	86	4.2	3.9	170	11.4%	0.55 [0.29, 0.82]
Fang Ran 2020	10.7	5.3	82	6.2	2.7	115	11.1%	1.12 [0.82, 1.43]
Jian Guo 2018	21.4	17.2	326	20.4	17.6	790	12.3%	0.06 [-0.07, 0.19]
Le Hou 2019	5.7	4.6	141	6.3	6.5	120	11.6%	-0.11 [-0.35, 0.14]
Lei Mao 2020	4.8	4.6	72	6.2	7.1	116	11.2%	-0.22 [-0.52, 0.07]
M.L Alexandrova 2016	17.5	24.5	20	1.9	1.1	11	6.7%	0.77 [0.00, 1.53]
Mingsi Zhang 2022	1.4	2.5	105	0.9	2.5	82	11.2%	0.20 [-0.09, 0.49]
Zhengbao Zhu 2019	2.6	3	340	2.2	2.6	298	12.2%	0.14 [-0.01, 0.30]
**Subtotal (95% CI)**			**1172**			**1702**	**87.7%**	**0.27 [0.02, 0.53]**
Heterogeneity: Tau² = 0.11; Chi² = 62.88, df = 7 (P < 0.00001); I² = 89%								
Test for overall effect: Z = 2.11 (P = 0.03)								
**1.4.2 Retrospective study**								
He Jia 2020	6.7	4.6	523	3.2	4.1	496	12.3%	0.80 [0.67, 0.93]
**Subtotal (95% CI)**			**523**			**496**	**12.3%**	**0.80 [0.67, 0.93]**
Heterogeneity: Not applicable								
Test for overall effect: Z = 12.30 (P < 0.00001)								
**Total (95% CI)**			**1695**			**2198**	**100.0%**	**0.35 [0.06, 0.64]**
Heterogeneity: Tau² = 0.17; Chi² = 131.77, df = 8 (P < 0.00001); I² = 94%								
Test for overall effect: Z = 2.35 (P = 0.02)								
Test for subgroup differences: Chi² = 13.19, df = 1 (P = 0.0003), I² = 92.4%								

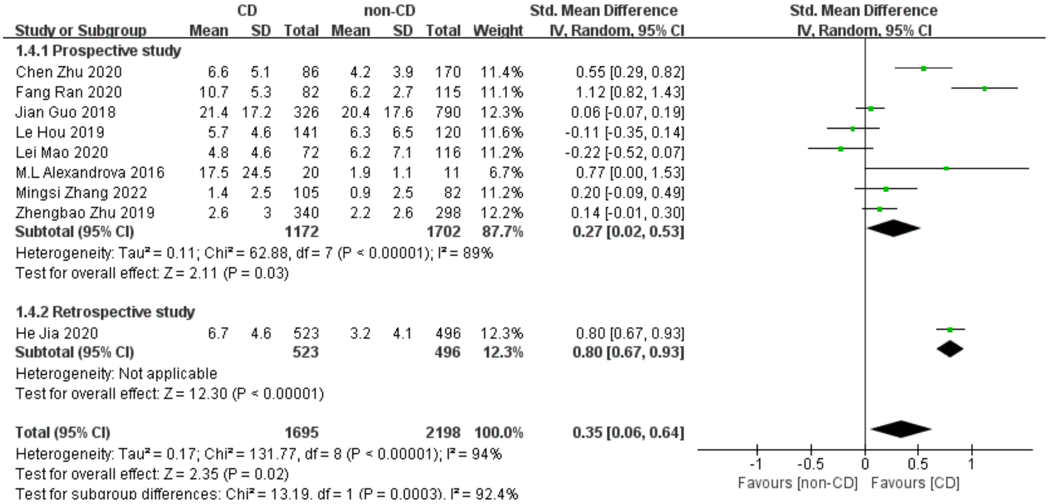

Forest map of sample size

Study or Subgroup	CD Mean	CD SD	CD Total	non-CD Mean	non-CD SD	non-CD Total	Weight	Std. Mean Difference IV, Random, 95% CI
**1.5.1 N<500**								
Chen Zhu 2020	6.6	5.1	86	4.2	3.9	170	11.4%	0.55 [0.29, 0.82]
Fang Ran 2020	10.7	5.3	82	6.2	2.7	115	11.1%	1.12 [0.82, 1.43]
Le Hou 2019	5.7	4.6	141	6.3	6.5	120	11.6%	-0.11 [-0.35, 0.14]
Lei Mao 2020	4.8	4.6	72	6.2	7.1	116	11.2%	-0.22 [-0.52, 0.07]
M.L Alexandrova 2016	17.5	24.5	20	1.9	1.1	11	6.7%	0.77 [0.00, 1.53]
Mingsi Zhang 2022	1.4	2.5	105	0.9	2.5	82	11.2%	0.20 [-0.09, 0.49]
**Subtotal (95% CI)**			**506**			**614**	**63.2%**	**0.36 [-0.07, 0.79]**
Heterogeneity: Tau² = 0.25; Chi² = 56.22, df = 5 (P < 0.00001); I² = 91%								
Test for overall effect: Z = 1.66 (P = 0.10)								
**1.5.2 N≥500**								
He Jia 2020	6.7	4.6	523	3.2	4.1	496	12.3%	0.80 [0.67, 0.93]
Jian Guo 2018	21.4	17.2	326	20.4	17.6	790	12.3%	0.06 [-0.07, 0.19]
Zhengbao Zhu 2019	2.6	3	340	2.2	2.6	298	12.2%	0.14 [-0.01, 0.30]
**Subtotal (95% CI)**			**1189**			**1584**	**36.8%**	**0.33 [-0.15, 0.82]**
Heterogeneity: Tau² = 0.18; Chi² = 74.62, df = 2 (P < 0.00001); I² = 97%								
Test for overall effect: Z = 1.36 (P = 0.17)								
**Total (95% CI)**			**1695**			**2198**	**100.0%**	**0.35 [0.06, 0.64]**
Heterogeneity: Tau² = 0.17; Chi² = 131.77, df = 8 (P < 0.00001); I² = 94%								
Test for overall effect: Z = 2.35 (P = 0.02)								
Test for subgroup differences: Chi² = 0.01, df = 1 (P = 0.94), I² = 0%								

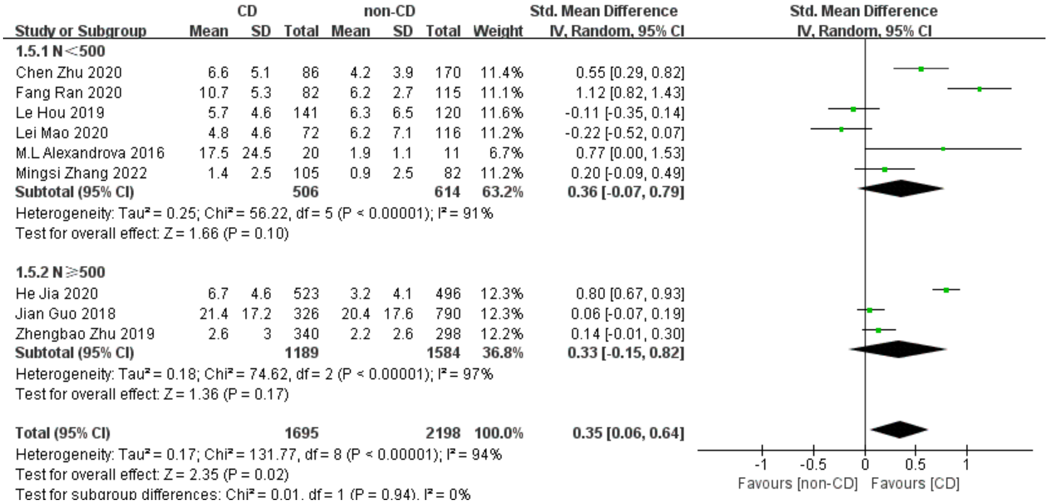

Forest map of source of CRP

Study or Subgroup	CD Mean	CD SD	CD Total	non-CD Mean	non-CD SD	non-CD Total	Weight	Std. Mean Difference IV, Random, 95% CI
**1.1.1 serum**								
Fang Ran 2020	10.7	5.3	82	6.2	2.7	115	11.1%	1.12 [0.82, 1.43]
He Jia 2020	6.7	4.6	523	3.2	4.1	496	12.3%	0.80 [0.67, 0.93]
Jian Guo 2018	21.4	17.2	326	20.4	17.6	790	12.3%	0.06 [-0.07, 0.19]
Le Hou 2019	5.7	4.6	141	6.3	6.5	120	11.6%	-0.11 [-0.35, 0.14]
Lei Mao 2020	4.8	4.6	72	6.2	7.1	116	11.2%	-0.22 [-0.52, 0.07]
M.L Alexandrova 2016	17.5	24.5	20	1.9	1.1	11	6.7%	0.77 [0.00, 1.53]
Mingsi Zhang 2022	1.4	2.5	105	0.9	2.5	82	11.2%	0.20 [-0.09, 0.49]
Zhengbao Zhu 2019	2.6	3	340	2.2	2.6	298	12.2%	0.14 [-0.01, 0.30]
**Subtotal (95% CI)**			**1609**			**2028**	**88.6%**	**0.32 [0.00, 0.64]**
Heterogeneity: $Tau^2 = 0.19; Chi^2 = 129.09, df = 7 (P < 0.00001); I^2 = 95\%$								
Test for overall effect: Z = 1.98 (P = 0.05)								
**1.1.2 plasma**								
Chen Zhu 2020	6.6	5.1	86	4.2	3.9	170	11.4%	0.55 [0.29, 0.82]
**Subtotal (95% CI)**			**86**			**170**	**11.4%**	**0.55 [0.29, 0.82]**
Heterogeneity: Not applicable								
Test for overall effect: Z = 4.10 (P < 0.0001)								
**Total (95% CI)**			**1695**			**2198**	**100.0%**	**0.35 [0.06, 0.64]**
Heterogeneity: $Tau^2 = 0.17; Chi^2 = 131.77, df = 8 (P < 0.00001); I^2 = 94\%$								
Test for overall effect: Z = 2.35 (P = 0.02)								
Test for subgroup differences: $Chi^2 = 1.19, df = 1 (P = 0.28), I^2 = 15.8\%$								

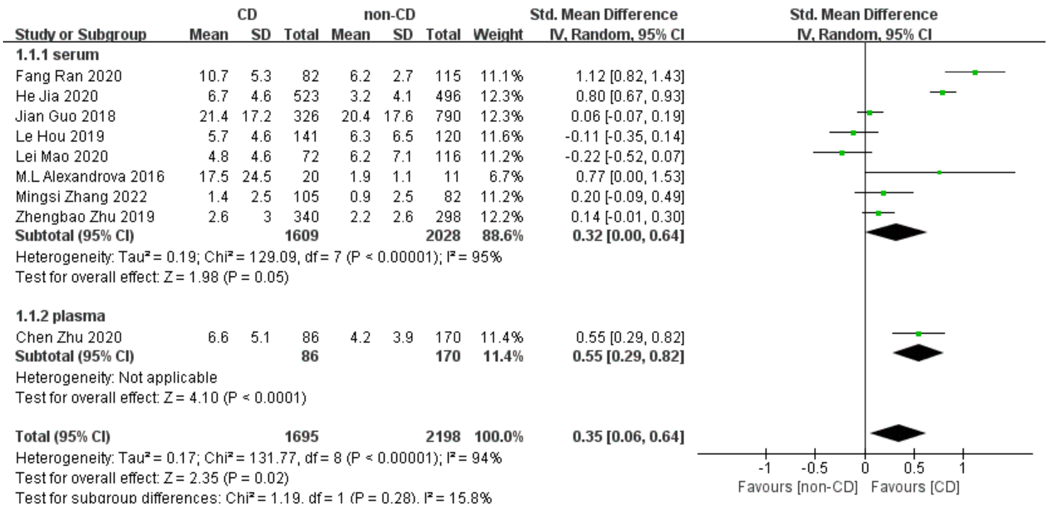

Supplement: Supplementary file 1 [file brainsci-13-01082-s001.zip › brainsci-2466950-supplementary-updated.pdf]
